# Diversity of duckweed (*Lemnaceae*) associated yeasts and their plant growth promoting characteristics

**DOI:** 10.3934/microbiol.2023026

**Published:** 2023-05-16

**Authors:** Napapohn Kajadpai, Jirameth Angchuan, Pannida Khunnamwong, Nantana Srisuk

**Affiliations:** 1 Department of Microbiology, Faculty of Science, Kasetsart University, Bangkok, 10900, Thailand; 2 Biodiversity Center Kasetsart University (BDCKU), Bangkok 10900, Thailand

**Keywords:** yeast, diversity, duckweed, plant growth promotion

## Abstract

The diversity of duckweed (*Lemnaceae*) associated yeasts was studied using a culture-dependent method. A total of 252 yeast strains were isolated from 53 duckweed samples out of the 72 samples collected from 16 provinces in Thailand. Yeast identification was conducted based on the D1/D2 region of the large subunit (LSU) rRNA gene sequence analysis. It revealed that 55.2% and 44.8% yeast species were Ascomycota and Basidiomycota duckweed associated yeasts, respectively. Among all, *Papiliotrema laurentii*, a basidiomycetous yeast, was found as the most prevalent species showing a relative of frequency and frequency of occurrence of 21.8% and 25%, respectively. In this study, high diversity index values were shown, indicated by the Shannon-Wiener index (*H′*), Shannon equitability index (*E_H_*) and Simpson diversity index (*1-D*) values of 3.48, 0.86 and 0.96, respectively. The present results revealed that the yeast community on duckweed had increased species diversity, with evenness among species. Principal coordinate analysis (PCoA) revealed no marked differences in yeast communities among duckweed genera. The species accumulation curve showed that the observed species richness was lower than expected. Investigation of the plant growth promoting traits of the isolated yeast on duckweed revealed that 178 yeast strains produced indole-3-acetic acid (IAA) at levels ranging from 0.08–688.93 mg/L. Moreover, siderophore production and phosphate solubilization were also studied. One hundred and seventy-three yeast strains produced siderophores and exhibited siderophores that showed 0.94–2.55 activity units (AU). One hundred six yeast strains showed phosphate solubilization activity, expressed as solubilization efficiency (SE) units, in the range of 0.32–2.13 SE. This work indicates that duckweed associated yeast is a potential microbial resource that can be used for plant growth promotion.

## 1. Introduction

Duckweed is a small and fast growing aquatic plant in the *Lemnaceae* family, which has 38 species in five genera, *Landotia*, *Lemna*, *Spirodela*, *Wolffia*, and *Wolffiella* [1]. They are distributed around the world and can tolerate polluted water [2]. Four of the duckweed species are found globally. However, *Wolffiella* is only species found in the Americas and Africa [3]. Due to their aquatic habitat, duckweed is easy to harvest and requires no arable land.

Duckweed is a well-known feed for livestock such as ducks, swine, chicken, and fish due to its high protein content alongside other nutrients such as vitamins and astaxanthin [4]. Moreover, duckweed, especially *Wolffia*, has long been used in traditional Asian foods in countries such as Thailand, Laos, and Cambodia [5]. The nutritional content of duckweed in terms of starch, protein, fat, minerals, vitamins, and phytosterol content, as well as amino acid and fatty acids profiles has been analyzed [6]–[8]. Results suggested that duckweed contains high value nutrients and its use is recommended by the World Health Organization (WHO). Products derived from *Lemna* and *Wolffia* species have been deemed Generally Recognized as Safe (GRAS) by the US Food and Drug Administration. Over the past decade, several companies (e.g., Parabel, Hinoman, GreenOnyx) have been established to develop duckweed as a food and protein source [9]. Additionally, the fast-growing character and chemical composition of duckweed makes it a promising energy resource.

Duckweed directly absorbs nutrients from water. Therefore, it can be used as a phytoremediation agent for waste water treatment [10]. Duckweed removes nitrogenous compounds [11] as well as heavy metals by uptake through their root fronds [12],[13]. Wetlands contaminated with hazardous chemicals can also be remediated using duckweed [14]. More recently, application of duckweed to remediate crude oil contaminants and polyester manufacturing effluents from wastewater have been reported [15],[16].

Duckweed uptakes nutrients (nitrogen and phosphorus) from waste water to support its growth and to store nutrients in its tissue. When the nutrients are completely removed from waste water, duckweed uses internally stored nutrients to support their growth for a period of time. Duckweed has the capability to accumulate starch at levels of up to 50% of its dry weight and further increases starch accumulation during waste water treatment [17]. Several treatments can induce starch accumulation in duckweed, such as abiotic stressors and nutrient limitations [18],[19]. This enables utilization of duckweed for bioenergy production [20]. Starch in duckweed can be hydrolyzed to sugars, which can consequently be fermented to alcohols such as ethanol and butanol [18]. Bioenergy production from duckweed is more feasible than that from other energy plants, such as sugarcane, since their cell walls contain less lignin. Therefore, starch can easily be pooled and converted to fermentable sugars [21],[22] prior to biofuel production. Moreover, the duckweed biomass can be used to produce biogas by anaerobic digestion [23].

Plant associated yeasts are those that colonize either inside plant tissue or on surface of host plant. This relationship between plants and yeasts is mutualistic [24]. The plant provides some nutrients and a stable environment for yeasts, while the yeasts produce metabolites that promote the plants resistance to unfavorable conditions and phytopathogens. Moreover, plant associated yeasts have capabilities to promote plant growth by production of phytohormones such as indole-3-acetic acid, siderophores, and 1-aminocyclopropane-1-carboxylic acid (ACC) deaminase. They also solubilize phosphate and zinc, as well as present antagonistic activities against plant pathogens [25],[26].

Research on duckweed associated microorganisms has been reported [27]–[30]. However, to the best of our knowledge, a study of duckweed associated yeasts has not been previously performed. Therefore, this study aims to investigate yeast communities associated with duckweed (*Lemnaceae*) using culture techniques together with molecular yeast identification. A species accumulation curve was investigated and diversity indices were evaluated. The isolated yeasts were screened for plant growth promoting factors, including indole-3-acetic acid and siderophore production, as well as phosphate solubilization.

## 2. Materials and methods

### 2.1. Sample collection and yeast isolation

A total of 72 duckweed samples were collected from 28 districts in 16 provinces of Thailand between February 2021 and May 2022. The samples were collected and kept in plastic bags during transport to the laboratory. Duckweed samples were identified to genus level by eye observation of morphological characteristics [31]. Yeast isolation was carried out within three days of collection.

Yeasts were isolated directly. Approximately 1 g of duckweed was rinsed with a sterile normal saline solution (NSS, 0.85% NaCl) to remove dirt. Then, the samples were put into 250 mL Erlenmeyer flasks containing 100 mL of sterile NSS, followed by shaking at 100 rpm for 30 min. This was repeated twice for 10 min to remove the microorganisms contaminated with water from the sampling site. The effectiveness of the surface wash was verified by spreading 0.1 mL of the rinse solution onto yeast extract-malt extract (YM) agar (0.3% yeast extract, 0.3% malt extract, 0.5% peptone, 1.0% dextrose and 1.5% agar) in Petri dishes. If no microbial colonies appeared after incubation at 30 °C for seven days, the microorganisms contaminated with water from the sampling site were completely removed. After the surface wash, duckweed samples were ground using a sterile mortar and pestle with 0.3 mL of sterile NSS. Then, homogenized samples were spread onto YM agar supplemented with 0.12% sodium propionate and 0.1% chloramphenicol to prevent filamentous fungi and bacterial contamination, respectively. Plates were incubated at 30 ± 2 °C for either 2–7 days or until yeast colonies appeared. Yeast colonies with different morphologies were cross-streaked onto YM agar for purification. Purified yeast isolates were stored at −20 °C in YM broth supplemented with 30% (v/v) glycerol for long term preservation.

### 2.2. Yeast identification

The DNA of purified yeasts was extracted according to Ruiz-Barba et al. [32] with slight modifications. Yeast cells grown on YM agar for 18–24 h were suspended in 100 µL of sterile deionized water in a sterile 1.5 mL plastic microtube. Then, an aliquot (100 µL) of chloroform/isoamyl alcohol (24:1) was added to the suspension prior to vortexing for 5 min. The mixtures were centrifuged at 14,000 × g for 5 min. An aliquot of the upper aqueous phase was used as a DNA template for amplification. The D1/D2 domain of the large subunit (LSU) rRNA gene was amplified using a polymerase chain reaction (PCR) with NL1 and NL4 primers [33]. The PCR products were examined using agarose gel electrophoresis under blue light and compared with DNA markers. Then, the PCR products were purified with a FavorPrep™ GEL/PCR Purification Mini kit (Favorgen, Austria). The purified PCR products were sent for DNA sequencing at First BASE Laboratories, Malaysia. The sequences were compared with those in the GenBank (http://www.ncbi.nlm.nih.gov/) database using a nucleotide BLASTN search [34]. The criteria of yeast species identification using similarity of the D1/D2 region of the LSU rRNA gene sequence were 99.41% and 99.51% for ascomycetous and basidiomycetous yeasts, respectively. The criterion for distinguish yeast genera using the similarity of D1/D2 region of LSU rRNA gene sequence was 97.11% [35].

### 2.3. Phylogenetic analysis

Phylogenetic analysis was performed based on the sequence of the D1/D2 region of the LSU rRNA gene to confirm the yeast identification using the MEGA (Version 7.0.26) program. The sequence of a representative yeast from an individual species was subjected to alignment with the type strain sequences from GenBank. Then, the alignment was used for phylogenetic tree construction. A phylogenetic tree was built from the evolutionary distance using a GTR evolutionary model and the maximum-likelihood method. Bootstrap values were calculated from 1000 replicates.

### 2.4. Biodiversity analysis

Yeast isolates that showed identical DNA sequences were excluded from the collection for analysis of biodiversity indices, species richness, and for principal coordinate analysis (PCoA). Yeast diversity was analyzed using the Shannon Wiener index (*H′*). Yeast community evenness was analyzed with Shannon equitability index (*E_H_*) [36],[37], which assumes a value between 0 and 1; a value approaching 1 indicates complete evenness among the species, while a value approaching 0 indicates no evenness. The equations are as follows:

$$Shannon-Wiener\;index\;(H\prime )=-\overset{S}{\underset{i=0}{\sum }}Pi(lnPi)$$ (1)

$$Shannon\;equitability\;index\;(EH)=\frac{H\prime }{\text{lnS}}$$ (2)

where, *Pi* is the proportion of each species in the sample and S is the total number of species in the total sample.

The Simpson diversity index (*1-D*) is a measure of diversity that considers both richness and evenness. This value is between 0 and 1, where 1 represents infinite diversity and 0 is no diversity [38]. Its equation is as follows:

$$Simpson\;diversity\;index\;(1-D)=1-\frac{\sum^{\text{​}}n_{i}\left(n_{i}-1\right)}{N\left(N-1\right)}$$ (3)

where, *n_i_* is the number of strains of each species and *N* is the total number of strains of all species.

The frequency of occurrence (%) was calculated as the number of samples in which a particular species was observed divided by the total number of samples. The relative frequency (%) was calculated as the number of strains of an individual species as a proportion of the total number of strains.

The species richness was estimated using the EstimateS software, Version 9.1 which calculated species richness from the sampling effort of the Chao 1, Jack 1, and bootstrap estimators with sample-based abundance data (i.e., the classic EstimateS input) [39].

The similarity of yeast communities associated with duckweed was measured using PCoA based on Jaccard similarity indices. Computational analysis was performed using PAST software, Version 4.0 [40].

### 2.5. Plant growth promoting factors

#### 2.5.1. Indole-3-acetic acid production

Yeast was inoculated into 3 mL of yeast extract peptone dextrose medium (YPD) containing 1% yeast extract, 2% peptone, and 2% dextrose and incubated at 30 °C and 200 rpm for 16–18 h. Then, the yeast inoculum was transferred to a 125 mL Erlenmeyer flask containing 25 mL of YPD medium supplemented with 0.1% L-tryptophan. The initial OD_600_ was adjusted to 0.2 prior to incubation on a rotary shaker at 30 °C and 170 rpm for 3 days. Final OD_600_ values were spectrophotometrically determined. The samples were centrifuged at 14,000 × g for 5 min and the supernatants were collected for IAA analysis using high performance liquid chromatography (HPLC; Nexera LC-40 series, Shimadzu, Japan) with a Cosmosil SC18-MS-II column (Nacalai Tesque, Japan) and UV detector at 280 nm. A mixture of ethanol, acetic acid, and water (60:1.5:40 v/v/v) was used as a mobile phase with a flow rate of 0.3 mL/min, as described by Nutaratat et al. [41]. Authentic IAA (Sigma, USA) was used as a standard.

#### 2.5.2. Siderophore production and phosphate solubilization

A yeast inoculum was prepared on a YPD medium supplement with 1.5% agar at 30 °C for 24 h. Then, the yeast inoculum was point inoculated on Chrome-Azurol S (CAS) agar [42] and Pikovskaya's agar [43] to determine siderophore production and phosphate solubilization, respectively, prior to incubation at 30 °C for seven days. Siderophore activity unit (AU) values were calculated as a ratio between the diameter of an orange halo zone and that of its associated colony. The phosphate solubilization efficiency (SE) was calculated as a ratio of the diameter of a clear zone to that of its associated colony.

## 3. Results

### 3.1. Sample collection and yeast isolation

Two-hundred and fifty-two yeast strains were isolated from 53 duckweed samples out of a total 72 samples collected from 16 Thai provinces in 2021–2022. The results indicated that 183, 36, 23, and 10 yeast strains were isolated from 33 *Lemna*, 9 *Spirodela*, 8 *Landotia*, and 3 *Wolffia* samples, corresponding to 45.83%, 12.5%, 11.11%, and 4.17% of individual duckweed genera associated with yeast, respectively (Table S1). The effectiveness of the sample surface wash procedure was tested by spreading the final rinse water onto YM agar plates. A few fungal colonies were found in the final rinse water of 3 out of 72 samples after seven days of incubation. However, no yeast or bacterial colonies were found in the final rinse water for any of the samples.

### 3.2. Yeast identification

**Figure 1.**
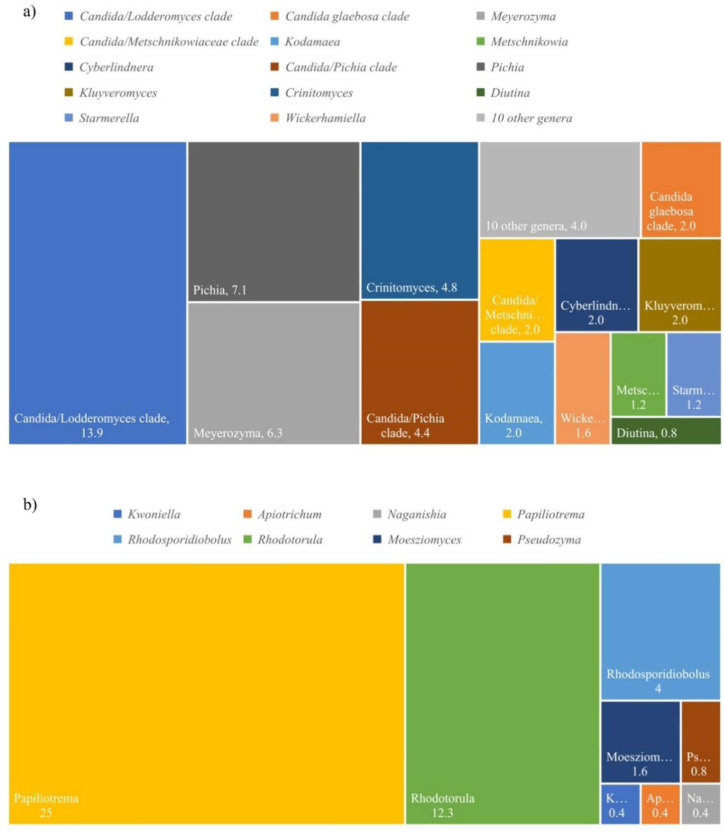
Percentage of yeast genera found in duckweed samples, a) yeast in the phylum Ascomycota, b) yeast in the phylum Basidiomycota.

Two-hundred and fifty-two yeast strains were identified based on the D1/D2 region of the LSU rRNA gene sequence. According to Vu et al. [35], there is an increased number of yeast in the phylum Ascomycota (55.2%) when compared to Basidiomycota (44.8%). The proportion of yeast genera in duckweed samples is shown in Figure 1, whereas the relative of frequency data of each species is shown in Table S2.

Two-hundred and thirty-seven yeasts out of 252 strains were identified as yeast species and 15 strains were identified to the genus level. There were four genera and four groups of *Candida*, including one strain of *Candida* sp. Group 1 (closely related to *Candida tropicalis* in the *Candida/Lodderomyces* clade), two strains of *Candida* sp. Group 2 (closely related to *C. suratensis* in the *Candida*/*Metschnikowiaceae* clade), one strain of *Candida* sp. Group 3 (closely related to *C. yuanshanica* in the *Candida/Wickerhamomyces* clade), one strain of *Candida* sp. Group 4 (closely related to *C. pseudolambica* in the *Pichia/Candida* clade), three strains of a *Starmerella* sp. (closely related to *Starmerella caucasica*), one strain of a *Zygoascus* sp. (closely related to *Zygoascus polysorbophila*), five strains of *Papiliotrema* sp. (closely related to *Papiliotrema laurentii*), and one strain of *Rhodotorula* sp. (closely related to *Rhodotorula toruloides*) (Table S3). These 15 yeast strains showed nucleotide sequence similarities that ranged between 97.11% and 99.41% to their closest species in the GenBank database.

**Figure 2.**
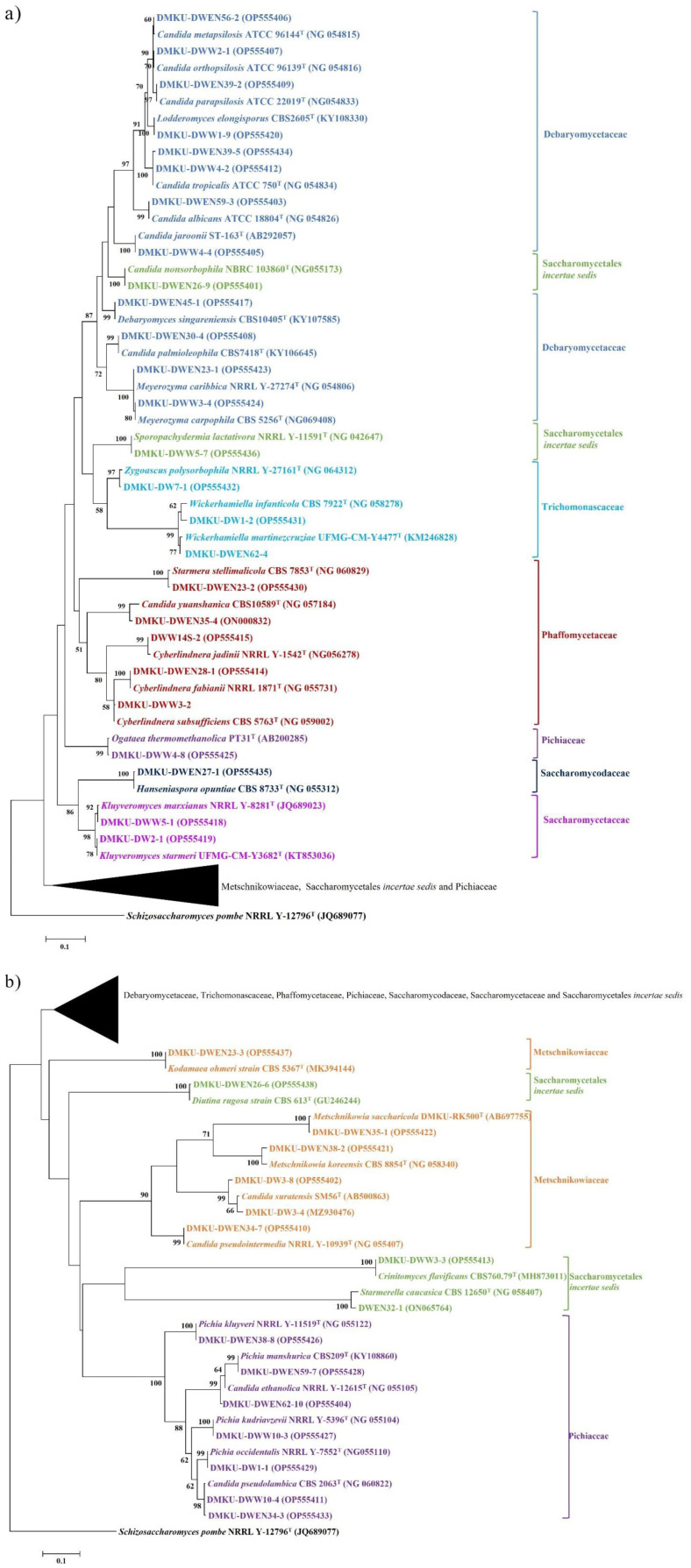
Phylogenetic placement of known species of the representative yeast species from duckweed (phylum Ascomycota) based on sequences of the D1/D2 region of the LSU rRNA gene. Reference sequences retrieved from the GenBank database are included. The tree was constructed with the maximum-likelihood method and the GTR evolutionary model. Numbers on the branches represent the bootstrap values (>50%) from 1000 random replicates. The scale bar corresponds to a genetic distance of 0.1 substitutions per position. *Schizosaccharomyces pombe* NRRL Y-12796^T^ (JQ689077) was used as an outgroup in this analysis. a) A part of the tree showing the phylogenetic relationships of a partial taxa within Debaryomycetaceae, Saccharomycetales *incertae sedis*, Trichomonascaceae, Phaffomycetaceae, Pichiaceae, Saccharomycodaceae and Saccharomycetaceae b) Part of the tree that shows the phylogenetic relationships of another partial taxa within Metschnikowiaceae, Saccharomycetales *incertae sedis* and Pichiaceae.

**Figure 3.**
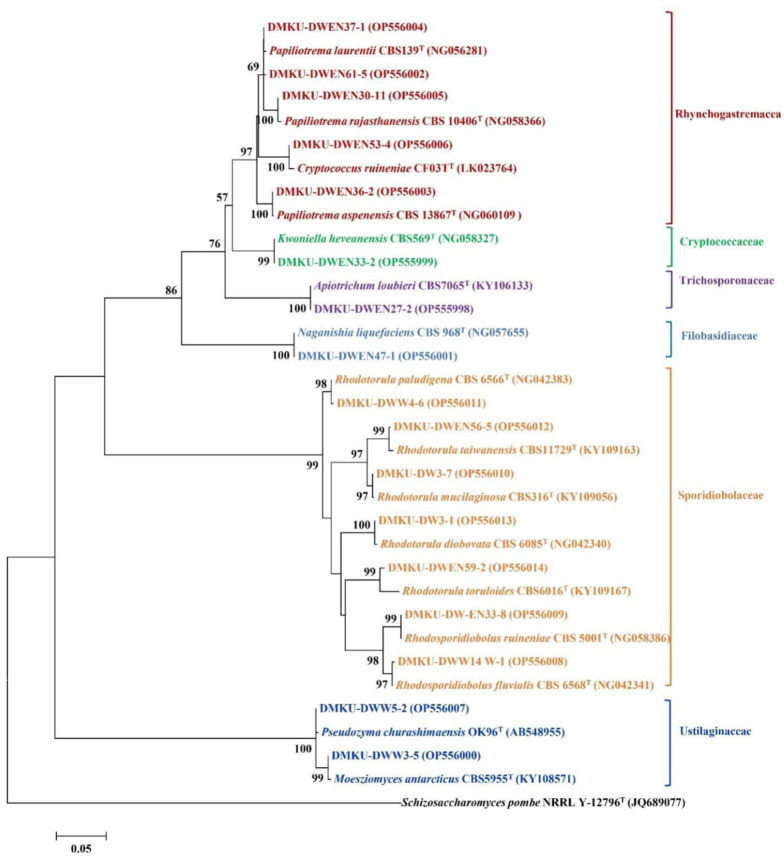
Phylogenetic placement of known species of the representative yeast species from duckweed (phylum Basidiomycota) based on the sequence of the D1/D2 region of the LSU rRNA gene. Reference sequences retrieved from the GenBank database are included. The tree was constructed with the maximum-likelihood method and the GTR evolutionary model. Numbers on the branches represent the bootstrap values (>50%) from 1000 random replicates. The scale bar corresponds to a genetic distance of 0.05 substitutions per position. *Schizosaccharomyces pombe* NRRL Y-12796^T^ (JQ689077) was used as an outgroup in this analysis.

Among the 237 strains of known yeast species, 130 strains were identified as 35 known members of eight families in the phylum Ascomycota, including Debaryomycetaceae (11 species, 58 strains), Metschnikowiaceae (4 species, 11 strains), Phaffomycetaceae (4 species, 6 strains), Pichiaceae (7 species, 29 strains), Saccharomycetaceae (2 species, 5 strains), Saccharomycodaceae (1 species, 1 strain), Trichomonascaceae (2 species, 4 strains), and Saccharomycetales *incertae sedis* (4 species, 16 strains). However, 107 strains were identified as 15 known members of six families and three subphyla in the phylum Basidiomycota, including Filobasidiaceae (1 species, 1 strain), Cryptococcaceae (1 species, 1 strain), Rhynchogastremaceae (4 species, 58 strains), Trichosporonaceae (1 species, 1 strain), Sporidiobolaceae (6 species, 40 strains), and Ustilaginaceae (2 species, 6 strains). The phylogenetic placement of representative yeast isolated from duckweed is shown in Figure 2 (phylum Ascomycota) and Figure 3 (phylum Basidiomycota). The representative sequence data of those yeasts showing the highest similarity of the D1/D2 region of the LSU rRNA gene to the corresponding type strains were submitted to the GenBank database under the following accession numbers: ON065764, ON000832, MZ930476, OP609955, OP555401-OP555438, and OP555998-OP556014.

### 3.3. Yeast diversity

The results shown in Figures 2 and 3 indicate a slightly higher number of yeast strains in the phylum Ascomycota than in Basidiomycota. However, the most abundant species in this study was *Papiliotrema laurentii* (55 strains out of 252 strains, which is equivalent to a 21.8% relative frequency). Additionally, the highest occurring species was *Pa. laurentii*, found in 18 out of 72 samples (25% frequency of occurrence; FO), followed by *Crinitomyces flavificans* (16.7% FO), *Candida tropicalis* (15.3% FO), and *Rhodotorula mucilaginosa* (13.9% FO). The other species had frequency of occurrence values between 1.4–9.7% (Table S2). Moreover, two yeast species, *Pa. laurentii* and *Cr. flavificans*, were found in all four genera of duckweed investigated in the present study.

**Figure 4.**
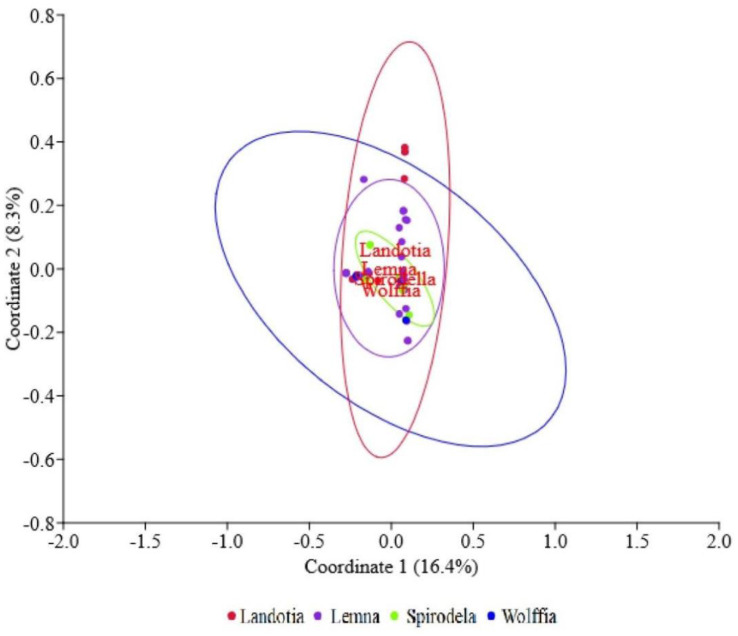
Principal Coordinate Analysis (PCoA) plots of yeast communities on duckweed samples using Jaccard similarity coefficient.

From 8 samples of *Landotia* duckweeds, 11 yeast species were found (members of 9 yeast genera in 6 families), whereas from 33 samples of *Lemna* duckweeds, 46 yeast species were found (members of 29 yeast genera in 13 families). In the case of *Spirodella* duckweeds, 20 yeast species (members of 14 yeast genera in 9 families) were found from 9 duckweed samples, while 5 yeast species (members of 5 yeast genera in 5 families) were found from 3 samples of *Wolffia* duckweeds. In order to determine difference in yeast diversity, the principal coordinate analysis (PCoA) was performed. As a result, no marked differences could be found in yeast species among duckweed genera (Figure 4). However, we observed that two yeast species, namely *Pa. laurentii* and *Cr. Flavificans*, can be isolated from all four duckweed genera.

**Figure 5.**
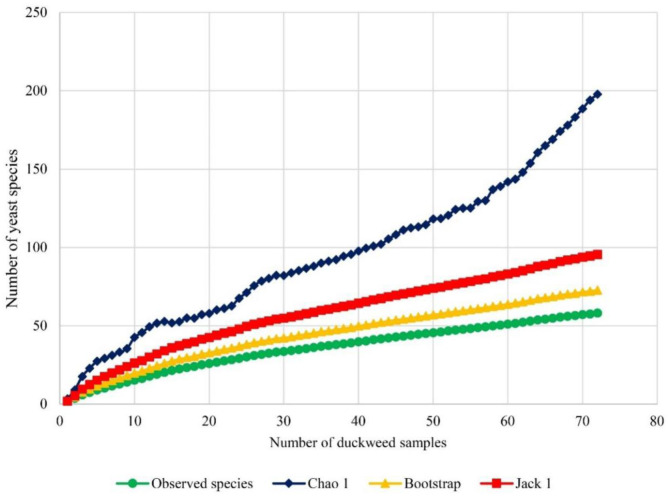
Species accumulation curves showing the relationship between the number of duckweed samples and the number of observed species. Chao 1, Jack 1 and bootstrap species richness estimators were plotted.

Diversity index values were calculated to evaluate the diversity of duckweed (*Lemnaceae*) associated yeasts. The Shannon-Wiener index (*H′*), Shannon equitability index (*E_H_*), and Simpson diversity index (*1-D*) values were 3.48, 0.86, and 0.96, respectively (Table 1). As the Shannon-Wiener index (*H′*) indicates diversity of yeast species, a higher value signifies an increased diversity of species in the habitat. If the index value is 0, only one species is present in the community. An *H′* value is usually between 1.5 and 3.5 [44]. The *H′* value obtained in the current study was 3.48. This value suggests that the yeast species from duckweed were highly diverse. The Shannon equitability index (*E_H_*) explains the evenness of species in a community. The term “evenness” simply refers to similarity of the abundance of different species in the community. An *E_H_* value is calculated between 0 and 1, and a value close to 1 indicates that the community has full evenness, while a value close to 0 means that there is no evenness among the species. The *E_H_* value of the present study was 0.86, revealing that yeasts species from duckweed in this community were highly even. The Simpson diversity index (*1-D*) is a measurement of diversity that is calculated from the number of species present, as well as the relative abundance of each species. When species richness and evenness increase, the diversity is greater. The value of the Simpson's diversity index in the present study was 0.96, which was close to 1, indicating that the yeast species from duckweed were highly rich and even. Estimation of the expected species richness demonstrated that the observed species richness was lower than the expected species richness (Figure 5). This result reveals that some yeast species may not have been recovered in this study.

**Table 1.** Diversity indices of yeast from duckweed.

Diversity indices	Values/Yeast name
Number of total samples	72
Number of total yeast strains	252
Total number of yeast species (*S*)	58
Shannon Weiner index (*H′*)	3.48
Shannon Equitability index (*E_H_*)	0.86
Simpson diversity index (*1-D*)	0.96
The most prevalent yeast species	*Papiliotrema laurentii*

### 3.4. Study of plant growth promoting factors

The obtained yeasts were studied to determine their plant growth promoting factors, including production of indole-3-acetic acid (IAA) and siderophores, as well as phosphate solubilization. The results are shown in Table 2.

One hundred seventy-eight yeast produced IAA at levels ranging from 0.08–688.93 mg/L. The highest IAA producing yeast in the present study was *Pichia occidentalis* DW1-1. Among these, 58, 36, and 85 yeast strains produced IAA in the ranges of 0.08–49.54, 50.13–99.93, and 100–688 mg/L, respectively. It is notable that five strains were found to produce higher than 500 mg/L of IAA in YPD broth with tryptophan supplementation.

One hundred seventy-three yeast strains produced siderophores, evaluated as siderophore activity units (AU), in the range of 0.94–2.55. *Rhodosporidiobolus ruineniae* DWEN33-8 showed the highest siderophore producing capability. From a total of 173 strains, 138 and 35 exhibited siderophore AU units in ranges of 0.94–1.94 and 2.00–2.55, respectively. However, 21 yeast strains did not grow on CAS agar after seven days of incubation.

The results revealed that 106 yeast strains exhibited phosphate solubilization activity, expressed as solubilization efficiency (SE) units, in the range of 0.32–2.13. Among 106 strains of phosphate solubilizing yeasts, 68 and 38 strains exhibited activities in range of 0.32–0.99 and 1–2.13 SE units, respectively. The highest phosphate solubilizing yeast in this study was *Pichia kluyveri* DWEN38-8.

**Table 2.** Production of IAA, siderophores and phosphate solubilization of the yeast isolated from duckweed.

Yeast species	Yeast strain code	IAA production (mg/L)	Siderophore production (AU)	Phosphate solubilization (SE)
*Candida albicans* (*Candida/Lodderomyces* clade)	DWEN59-3	117.07 ± 1.67	1.10 ± 0	1.06 ± 0.03
*Candida jaroonii* (*Yamadazyma* clade)	DWW4-4	151.35 ± 2.76	0	0
*Candida metapsilosis* (*Candida/Lodderomyces* clade)	DWEN56-2	0	0	0
*Candida orthopsilosis* (*Candida/Lodderomyces* clade)	DWW2-1	25.62 ± 3.21	1.04 ± 0.03	1.11 ± 0.13
	DWW2-2	24.07 ± 1.08	1.41 ± 0.31	1.08 ± 0
	DWW2-3	19.88 ± 8.13	1.08 ± 0.01	1.10 ± 0.09
	DWW2-4	26.59 ± 3.11	1.16 ± 0.07	0
*Candida palmioleophila* (*Candida glaebosa* clade)	DWEN30-4	nd	1.09 ± 0	0
	DWEN30-5	24.73 ± 1.17	1.20 ± 0	0
	DWEN30-6	52.68 ± 32.04	1.23 ± 0.06	0
	DWEN30-13	16.28 ± 0.46	1.31 ± 0.17	0
	DWEN56-10	225.28 ± 4.46	0	0
*Candida parapsilosis* (*Candida/Lodderomyces* clade)	DW8-4	3.39 ± 0.13	1.50 ± 0	0
	DWEN30-3	2.05 ± 2.90	1.67 ± 0	0.97 ± 0.05
	DWEN30-7	nd	2.00 ± 0	0.43 ± 0.37
	DWEN30-9	0.08 ± 0.12	2.00 ± 0	0.94 ± 0.05
	DWEN30-10	82.66 ± 3.27	1.83 ± 0.29	0.97 ± 0.05
	DWEN30-15	27.15 ± 0.54	-	0.63 ± 0.15
	DWEN39-1	76.5 ± 1.69	1.43 ± 0.06	0.44 ± 0.08
	DWEN39-2	128.05 ± 1.64	1.7 ± 0.26	0.65 ± 0.09
	DWEN62-9	0	1.31 ± 0.05	0.67 ± 0.12
*Candida tropicalis* (*Candida/Lodderomyces* clade)	DWW4-2	166.44 ± 5.43	0	0
	DW18-1	168.6 ± 2.05	1.44 ± 0.19	0.52 ± 0.06
	DW18-2	176.89 ± 0.63	1.75 ± 0	0.84 ± 0.07
	DWEN23-4	nd	1.28 ± 0.05	0.50 ± 0.04
	DWEN23-6	50.13 ± 4.8	1.50 ± 0	0.71 ± 0.08
	DWEN23-7	88.15 ± 43.97	1.49 ± 0.15	0.71 ± 0.06
	DWEN23-8	60.2 ± 3.54	1.50 ± 0	0.73 ± 0.04
	DWEN29-1	0	-	1.19 ± 0.02
	DWEN30-2	64.31 ± 0.18	2.00 ± 0	1.06 ± 0.05
	DWEN30-8	81.39 ± 1.17	1.67 ± 0.29	0.57 ± 0.1
	DWEN30-14	nd	1.50 ± 0	0.62 ± 0.06
	DWEN30-16	235.37 ± 8.6	1.83 ± 0.29	0.61 ± 0.05
	DWEN33-3	nd	1.33 ± 0	0.76 ± 0.07
	DWEN38-1	408.03 ± 37.79	-	0.67 ± 0
	DWEN49-1	133.84 ± 5.36	1.50 ± 0	0.86 ± 0.02
	DWEN54-2	127.48 ± 1.05	1.33 ± 0	0.77 ± 0.02
	DWEN56-1	174.64 ± 8.57	1.42 ± 0.14	0.77 ± 0.07
	DWEN62-1	115.49 ± 3.55	1.28 ± 0.10	0.55 ± 0.14
	DWEN62-6	122.75 ± 1.78	1.5 ± 0	0.67 ± 0.04
*Candida* sp. group 1 (closely related to *C. tropicalis* in *Candida*/*Lodderomyces* clade)	DWEN39-5	527.77 ± 5.41	1.50 ± 0	0.47 ± 0.05
*Debaryomyces singareniensis*	DWEN45-1	179.76 ± 30.68	0	0.78 ± 0.14
*Lodderomyces elongisporus*	DWW1-9	13.83 ± 0.27	1.5 ± 0.29	0
*Meyerozyma caribbica*	DWEN23-1	76.45 ± 11.95	1.53 ± 0.06	0.86 ± 0.04
	DWEN23-9	17.2 ± 0.11	1.28 ± 0.05	0.56 ± 0.05
	DWEN34-2	254.34 ± 7.87	1.5 ± 0	0
	DWEN34-4	45.14 ± 5.14	1.19 ± 0.02	0.51 ± 0.11
	DWEN34-5	39.86 ± 1.36	1.23 ± 0.03	0
	DWEN34-6	40.61 ± 0.75	1.19 ± 0.02	0.32 ± 0.01
	DWEN43-1	153.72 ± 16.31	0	1.05 ± 0.04
	DWEN53-2	494.8 ± 40.30	0	0
	DWEN60-3	128.34 ± 1.40	1.13 ± 0.02	0.75 ± 0.11
	DWEN61-2	0	1.4 ± 0	1.19 ± 0.17
	DWEN62-11	37.23 ± 3.17	1.17 ± 0	0.74 ± 0.03
*Meyerozyma carpophila*	DWW3-4	101.93 ± 5	0	0.67 ± 0.6
	DWW4-1	122.84 ± 3.12	0	0
	DWW4-9	125.61 ± 5.33	0	0
	DW3-2	173.89 ± 5.81	1.38 ± 0.11	0
	DWEN39-4	70.29 ± 1.03	1.37 ± 0.11	0.34 ± 0.06
*Candida pseudointermedia* (*Candida/Metschnikowiaceae* clade)	DWEN30-1	130.51 ± 6.9	1.13 ± 0	0.63 ± 0.1
	DWEN34-7	366.45 ± 0.04	1.37 ± 0.05	1.08 ± 0
	DWEN62-8	419.48 ± 15.87	1.33 ± 0.07	0.79 ± 0.04
*Candida* sp. group 2 (closely related to *Candida suratensis* in *Candida*/*Metschnikowiaceae* clade)	DW3-4	19.89 ± 0.56	1.17 ± 0	0
	DW3-8	85.62 ± 16.9	1.33 ± 0	0
*Kodamaea ohmeri*	DWEN23-3	nd	1.38 ± 0	1.00 ± 0
	DWEN23-5	nd	1.46 ± 0.07	0.65 ± 0.06
	DWEN38-6	5.12 ± 6.47	1.40 ± 0.05	0.77 ± 0.05
	DWEN38-9	0	1.67 ± 0	0.47 ± 0.15
	DWEN50-1	337.68 ± 3.46	1.53 ± 0.12	0.81 ± 0.04
*Metschnikowia koreensis*	DWEN38-2	67.65 ± 2.38	1.67 ± 0	0.65 ± 0.3
*Metschnikowia saccharicola*	DWEN35-1	14.52 ± 0.01	-	1.18 ± 0
	DWEN35-3	24.54 ± 0.49	2.00 ± 0	1.06 ± 0.19
*Candida* sp. group 3 (closely related to *Candida yuanshanica* in *Candida*/*Wickerhamomyces* clade)	DWEN35-4	47.90 ± 0.33	0	0.95 ± 0.08
*Cyberlindnera fabianii*	DWEN28-1	0	-	1.15 ± 0.14
	DWEN28-2	0	-	1.24 ± 0.13
*Cyberlindnera jadinii*	DWW14 S-2	0	0	1.41 ± 0.17
*Cyberlindnera subsufficiens*	DWW3-2	0	0	1.00 ± 0.14
	DWW3-8	10.86 ± 0.45	0	0
*Starmera stellimalicola*	DWEN23-2	0	-	1.13 ± 0.06
*Candida ethanolica* (*Candida*/*Pichia* clade)	DWEN62-10	43.16 ± 0.69	1.50 ± 0	0
	DWEN62-12	nd	0	0
	DWEN62-13	0	1.36 ± 0.13	1.00 ± 0
*Candida pseudolambica* (*Candida*/*Pichia* clade)	DWW3-1	40.39 ± 0.68	0	0
	DWW10-2	nd	nd	nd
	DWW10-4	97.06 ± 15.43	0	1.00 ± 0
	DWEN26-3	nd	1.17 ± 0	0
	DWEN35-2	52.30 ± 2.30	0	0
	DWEN39-3	137.94 ± 4.00	1.19 ± 0.12	0.60 ± 0.04
	DWEN49-2	0	-	0
*Candida* sp. group 4 (closely related to *Candida pseudolambica* in *Candida*/*Pichia* clade)	DWEN34-3	44.43 ± 3.80	1.58 ± 0.14	0.62 ± 0.08
*Ogataea thermomethanolica*	DWW4-8	92.56 ± 17.61	0	0
*Pichia kluyveri*	DWEN38-8	322.46 ± 17.87	-	2.13 ± 0.05
*Pichia kudriavzevii*	DWW10-3	51.28 ± 2.77	0	0
	DWW11-1	124.38 ± 6.63	0	0.72 ± 0.05
	DWEN26-7	17.79 ± 1.02	2.00 ± 0	1.00 ± 0
	DWEN54-3	82.89 ± 3.64	1.25 ± 0	0.93 ± 0.03
	DWEN56-7	46.82 ± 1.15	1.67 ± 0	1.03 ± 0.09
	DWEN56-11	46.78 ± 0.36	1.42 ± 0.14	0.94 ± 0.04
	DWEN59-4	45.76 ± 1.20	1.25 ± 0	0.88 ± 0.03
	DWEN59-5	56.25 ± 1.51	1.25 ± 0	0.86 ± 0.12
	DWEN59-6	155.65 ± 133.22	1.39 ± 0.10	0.84 ± 0.17
	DWEN59-8	13.39 ± 0.01	1.22 ± 0.03	0.94 ± 0.07
	DWEN60-1	0	1.25 ± 0	0.91 ± 0.08
	DWEN61-1	0	1.56 ± 0.19	0.99 ± 0.09
	DWEN62-2	30.38 ± 0.34	1.67 ± 0.14	1.02 ± 0.03
	DWEN62-5	31.28 ± 1.00	0.94 ± 0.82	0.97 ± 0.02
	DWEN62-7	49.54 ± 2.35	1.31 ± 0.05	0.82 ± 0.12
*Pichia manshurica*	DWEN59-7	11.75 ± 5.41	1.17 ± 0	0.68 ± 0.30
*Pichia occidentalis*	DW1-1	688.93 ± 24.76	1.50 ± 0	0
*Kluyveromyces marxianus*	DWW5-1	20.25 ± 0.68	0	0
	DWW5-11	30.32 ± 0.32	0	0
*Kluyveromyces starmeri*	DW2-1	69.63 ± 0.55	1.00 ± 0.87	1.19 ± 0.01
	DWEN26-4	181.24 ± 52.91	2.00 ± 0	1.37 ± 0.15
	DWEN26-5	5.63 ± 0.52	2.00 ± 0	1.91 ± 0.37
*Candida nonsorbophila* (*Candida*/*Saccharomycetales* clade)	DWEN26-9	274.15 ± 2.01	1.00 ± 0	1.00 ± 0
*Crinitomyces flavificans*	DWW3-3	36.77 ± 1.3	0	1.07 ± 0.39
	DWW3-6	nd	0	1.06 ± 0.33
	DWW4-7	38.34 ± 1.57	0	1.04 ± 0
	DWW5-3	nd	-	0.88 ± 0.03
	DWW5-6	nd	nd	nd
	DWW5-8	78.69 ± 62.34	-	0.98 ± 0.39
	DWW5-9	105.03 ± 32.6	-	1.14 ± 0.34
	DWW5-12	86.58 ± 65.39	-	1.15 ± 0.09
	DWW7-1	114.76 ± 11.59	0	1.15 ± 0.09
	DWW8-1	nd	0	1.01 ± 0.15
	DWW11-2	121.04 ± 3.19	-	0.98 ± 0.06
	DW1-3	133.29 ± 2.4	-	0.83 ± 0.04
*Diutina rugosa*	DWEN26-6	14.61 ± 0.17	2.00 ± 0	0
	DWEN53-3	15.12 ± 0.09	1.39 ± 0.10	0
*Sporopachydermia lactativora*	DWW5-7	71.47 ± 3.8	0	0
*Starmerella* sp. (closely related to *Starmerella caucasica*)	DWEN32-1	41.62 ± 1.13	1.47 ± 0.12	0.84 ± 0.01
	DWEN32-2	43.85 ± 1.12	1.50 ± 0	0.81 ± 0.08
	DWEN32-3	18.98 ± 9.18	1.42 ± 0.22	0.89 ± 0.1
*Hanseniaspora opuntiae*	DWEN27-1	0	nd	nd
*Wickerhamiella infanticola*	DW1-2	16.88 ± 18.84	1.50 ± 0	0
	DW3-3	20.51 ± 1.44	1.50 ± 0	0
	DWEN30-12	196.65 ± 281.78	0	0
*Wickerhamiella martinezcruziae*	DWEN62-4	0	1.39 ± 0.10	0
*Zygoascus* sp. (closely related to *Zygoascus polysorbophila*)	DW7-1	0	1.60 ± 0	1.26 ± 0.15
*Naganishia liquefaciens*	DWEN47-1	32.74 ± 39.54	0	0
*Kwoniella heveanensis*	DWEN33-2	15.15 ± 1.00	-	0
*Papiliotrema aspenensis*	DWEN36-2	22.68 ± 0.89	-	0
*Papiliotrema laurentii*	DWW1-8	14.23 ± 0.33	1.41 ± 0.16	1.24 ± 0.04
	DWW9-1	nd	1.45 ± 0.18	0
	DWW14 S-1	5.45 ± 0.77	1.17 ± 0	0
	DWW14 S-3	5.09 ± 6.18	1.19 ± 0.05	0
	DWW14 S-8	0	1.15 ± 0.02	0
	DWW14 W-2	0	1.00 ± 0.88	0
	DWW14 W-3	0	1.44 ± 0.1	0
	DW8-3	nd	nd	nd
	DW15-1	0	1.50 ± 0	0
	DW15-2	0	1.39 ± 0.19	0
	DWEN21-1	1.12 ± 0.11	2.17 ± 0.29	0
	DWEN21-2	nd	nd	nd
	DWEN22-3	nd	1.89 ± 0.19	0
	DWEN22-4	1.51 ± 0.81	2.00 ± 0	0
	DWEN22-5	0	1.83 ± 0.29	0
	DWEN22-6	0	1.47 ± 0.21	0
	DWEN22-7	0	0	0
	DWEN22-8	0.97 ± 1.36	1.50 ± 0	0
	DWEN29-2	0	1.33 ± 0	0
	DWEN29-4	0	2.00 ± 0	0
	DWEN29-5	6.53 ± 0.05	2.00 ± 0	0
	DWEN29-6	nd	2.00 ± 0	0
	DWEN29-7	5.42 ± 1.07	1.83 ± 0.14	0
	DWEN29-8	6.64 ± 1.09	2.00 ± 0	0
	DWEN29-9	nd	2.00 ± 0	0
	DWEN29-10	0	2.00 ± 0	0
	DWEN29-11	nd	2.00 ± 0	0
	DWEN29-12	nd	2.00 ± 0	0
	DWEN31-1	26.45 ± 2.25	1.83 ± 0.14	0
	DWEN31-2	17.21 ± 0.56	1.75 ± 0	0
	DWEN31-3	24.92 ± 0.51	1.50 ± 0	0
	DWEN36-1	0	2.00 ± 0	0
	DWEN36-3	0	1.44 ± 0.1	0
	DWEN36-4	nd	1.50 ± 0	0
	DWEN36-5	nd	1.25 ± 0	0
	DWEN36-6	0	0	0
	DWEN36-7	0	1.50 ± 0	1.09 ± 0.01
	DWEN36-8	4.85 ± 6.86	2.00 ± 0	0
	DWEN37-1	14.99 ± 1.29	0	0.67 ± 0.21
	DWEN37-4	13.79 ± 0.97	0	0
	DWEN37-7	109.63 ± 8.05	0	0
	DWEN37-8	54.08 ± 0.45	2.00 ± 0	0.05 ± 0.08
	DWEN37-9	33.54 ± 5.14	2.00 ± 0	0
	DWEN37-10	79.38 ± 18.56	2.00 ± 0	0
	DWEN38-4	42.91 ± 22.87	1.44 ± 0.19	0
	DWEN38-5	2.31 ± 0.76	1.33 ± 0	0.56 ± 0.21
	DWEN38-7	49.09 ± 2.15	1.33 ± 0.33	0
	DWEN41-1	0	nd	nd
	DWEN41-2	0	2.08 ± 0.38	0
	DWEN41-4	47.77 ± 0.25	2.17 ± 0.29	0
	DWEN54-6	0	1.00 ± 0.87	0
	DWEN55-1	0	1.67 ± 0.29	0
	DWEN60-2	108.04 ± 0.45	1.31 ± 0.05	0
	DWEN60-4	78.43 ± 23.85	1.16 ± 0.15	0
	DWEN61-3	23.81 ± 19.01	1.19 ± 0.08	0
*Papiliotrema rajasthanensis*	DWEN30-11	74.25 ± 0.65	1.39 ± 0.1	0
*Papiliotrema ruineniae*	DWEN53-4	5.89 ± 0.30	0	0
*Papiliotrema* sp. (closely related to *Papiliotrema laurentii*)	DWW14 S-6	0	1.19 ± 0.05	0
	DWEN29-3	0	2.00 ± 0	0
	DWEN55-2	0	1.50 ± 0	0
	DWEN60-5	0	1.3 ± 0.09	0
	DWEN61-5	0	1.5 ± 0	0
*Apiotrichum loubieri*	DWEN27-2	0	0	0
*Rhodosporidiobolus fluvialis*	DWW14 S-4	277.08 ± 4.74	-	0
	DWW14 S-7	606.28 ± 92.19	0	0
	DWW14 W-1	562.11 ± 14.29	1.50 ± 0	0
	DWW14 W-4	663.10 ± 17.5	1.19 ± 0.13	0
*Rhodosporidiobolus ruineniae*	DW8-2	0	nd	nd
	DWEN26-10	50.63 ± 16.08	2.33 ± 0.29	1.22 ± 0.03
	DWEN33-1	nd	nd	nd
	DWEN33-5	73.73 ± 13.04	1.67 ± 0	0
	DWEN33-6	42.78 ± 38.98	1.61 ± 0.10	0
	DWEN33-8	50.75 ± 7.91	2.55 ± 0.45	0
*Rhodotorula mucilaginosa*	DW3-5	99.93 ± 3.01	-	0
	DW3-7	164.46 ± 10.19	-	0
	DW4-1	96.41 ± 0.47	-	0
	DW4-2	91.34 ± 0.19	1.50 ± 0.17	0
	DWEN22-1	24.46 ± 0.07	1.58 ± 0.07	0
	DWEN22-2	30.06 ± 2.24	2.00 ± 0	0
	DWEN39-6	386.8 ± 188.83	2.11 ± 0.19	0
	DWEN42-1	405.24 ± 130.15	2.22 ± 0.19	0
	DWEN43-2	307.03 ± 11.12	1.92 ± 0.14	0
	DWEN53-1	122.83 ± 2.46	1.94 ± 0.59	0
	DWEN53-5	102.44 ± 0.49	1.92 ± 0.14	0
	DWEN56-3	94.57 ± 0.94	1.78 ± 0.19	0
	DWEN56-6	109.11 ± 2.09	1.39 ± 0.10	0
	DWEN56-9	97.17 ± 15.38	0	0
	DWEN59-1	143.32 ± 10.22	1.61 ± 0.34	0
	DWEN62-3	0	1.89 ± 0.10	0
*Rhodotorula paludigena*	DWW4-6	nd	nd	nd
*Rhodotorula taiwanensis*	DWW5-5	nd	nd	nd
	DWW14 S-5	14.21 ± 5.07	0	0
	DWEN37-2	9.51 ± 4.54	0	0
	DWEN37-5	105.59 ± 31.58	0	0
	DWEN37-6	49.18 ± 0.19	0	0
	DWEN37-11	87.49 ± 16.72	2.00 ± 0	0
	DWEN41-3	73.29 ± 0.05	2.19 ± 0.17	0.36 ± 0.63
	DWEN55-3	11.14 ± 1.73	1.89 ± 0.19	0
	DWEN55-4	4.47 ± 0.06	1.67 ± 0.58	0
	DWEN55-6	24.62 ± 25.33	2.00 ± 0	0
	DWEN56-5	2.00 ± 0.16	1.50 ± 0.07	0
*Rhodotorula diobovata*	DW3-1	4.25 ± 3.18	1.89 ± 0.19	0
	DW3-9	9.84 ± 13.92	2.00 ± 0	0
*Rhodotorula* sp. (closely related to *Rhodotorula toruloides*)	DWEN59-2	29.57 ± 27.64	1.14 ± 0.01	0
*Moesziomyces antarcticus*	DWW3-5	101.93 ± 5	nd	nd
	DWW3-7	nd	nd	nd
	DWW4-3	nd	nd	nd
	DWW6-1	0	1.60 ± 0.17	0
*Pseudozyma churashimaensis*	DWW5-2	33.91 ± 33.4	1.61 ± 0.11	0
	DWW5-4	127.45 ± 3.19	0	0.93 ± 0.06

The results are expressed as mean ± standard deviation of duplicate of IAA production result, triplicate phosphate solubilization and siderophore production results. SE; The ratio of diameter of a clear zone and diameter of an associated colony, AU; The ratio of diameter of an orange halo zone and diameter of an associated colony. nd; The results are not available, -; Yeast that did not grow on CAS agar

## 4. Discussion

The *Lemnaceae* family consists of five genera, *Landotia*, *Lemna*, *Spirodella*, *Wolffia*, and *Wolffiella*. In the present study, four duckweed genera, *Landotia*, *Lemna*, *Spirodella*, and *Wolffia* were collected from 16 provinces in Thailand. It was not possible to obtain *Wolffiella* in Thailand since this duckweed genus is only found in the Americas and Africa [3]. Two-hundred and fifty-two yeasts were isolated from 53 samples of duckweed, but 19 of these samples contained no yeast species. The results of yeast identification and the proportion of yeast genera found in this study, shown in Figure 1, suggest a slightly higher number of yeasts in the phylum Ascomycota (55.2%) than those in Basidiomycota (44.8%). In accordance with this study, the ascomycetous yeast isolated from the inside of apple fruits (*Malus domestica*) and pear fruits (*Pyrus communis*) were more diverse than basidiomycetous yeast [45]. It was reported that the ascomycetous yeast belonging to the genera *Hanseniaspora* and *Metschnikowia* were predominant in the tissues of fleshly fruits such as chokeberry, hawthorn, pumpkin, euonymus, gooseberry, sea-buckthorn, honeysuckle, tomato, apple, plum, pear, oak, currant, brier, ash berries, and black haw [46]. Additionally, out of 98 yeast strains of 114 yeasts, the study of the phylloplane yeast of diverse plants in Thailand also showed that the majority were ascomycetous yeast [47]. In contrast, Khunnamwong et al. [48] reported increased numbers of basidiomycetous yeast when compared to ascomycetous yeast in the leaf tissue of the three crops, rice, corn, and sugarcane. The plant associated yeast species can either be ascomycetes or basidiomycetes. Relationships between yeast and plant species cannot yet be ruled out.

Thirty-five known yeast species in the phylum Ascomycota and 15 known species in the phylum Basidiomycota were identified in this study. Among these, 11 ascomycetous yeast species were reported as endophytic yeasts: *Candida metapsilosis* [48], *C. orthopsilosis* [49], *C. parapsilosis* [45],[50], *C. tropicalis* [48], *Meyerozyma caribbica* [51], *M. carpophila* [52], *C. pseudointermedia* [48], *Kodamaea ohmeri* [48], *Pichia kluyveri* [53], *Pi. kudriavzevii* [49], and *Hanseniaspora opuntiae* [53]. Among the basidiomycetous yeast, 11 species of the 15 known species were reported as endophytic yeasts: *Naganishia liquefaciens*, *Kwoniella heveanensis*, *Papiliotrema aspenensis*, *P. laurentii*, *P. rajasthanensis*, *Rhodosporidiobolus ruineniae* [48], *Rhodotorula mucilaginosa* [54], *R. paludigena*, *R. taiwanensis*, *Moesziomyces antarcticus*, and *Pseudozyma churashimaensis* [48].

Many yeast species were isolated from either plants, plant materials, or plant associated sources including fruits, exudates of plants, peat moss, phylloplane of plants, flowers, decomposed plants, fermented tea leaves (Miang), cacti, bark of trees, and cotton. These species include *C. albicans* [55], *C. jaroonii* [56],[57], *C. palmioleophila* [58], *Lodderomyces elongisporus* [59], *Metschnikowia koreensis* [57],[59]–[61], *M. saccharicola* [62], *Cyberlindnera fabianii* [57],[59], *Cy. jadinii* [63], *Cyberflaneur subsufficiens* [64], *Starmera stellimalicola* [65],[66], *C. ethanolica* [67], *C. pseudolambica* [57], *Pichia manshurica* [47], *P. occidentalis* [68], *Kluyveromyces marxianus* [47], *K. starmeri* [69], *C. nonsorbophila* [70], *Diutina rugosa* [57], *Wickerhamiella martinezcruziae* [71], *Papiliotrema ruineniae* [72], *Rhodosporidiobolus fluvialis* [73], and *Rhodotorula diobovata* [74].

Some yeast species obtained in the present study have not been previously reported as yeasts isolated from plants or plant materials, *viz*. *Debaryomyces singareniensis*, *Ogataea thermomethanolica*, *Crinitomyces flavificans*, *Sporopachydermia lactativora*, *Wickerhamiella infanticola*, and *Apiotrichum loubieri*. *De. singareniensis* was isolated from coal mine soil [75]. *Ogataea thermomethanolica* and *A. loubieri* were isolated from soil [76],[77]. *Crinitomyces flavificans* was isolated from food waste and river water [78]. *Sporopachydermia lactativora* was isolated from Antarctic seawater, buffalo feces, and has been reported as a contaminant yeast in wine production [77],[79],[80] while *Wickerhamiella infanticola* was isolated from insects in a vineyard, an infant ear, and fish intestines [81]–[83].

Fifteen yeast strains obtained during this study were identified to five genera including *Candida* sp. Group 1, *Candida* sp. Group 2, *Candida* sp. Group 3, *Candida* sp. Group 4, *Starmerella* sp., *Zygoascus* sp., *Papiliotrema* sp., and *Rhodotorula* sp. (Table S3). The plant species and environment are two factors that influence plant associated yeast diversity and community. In this study, duckweed habitats were aquatic. Therefore pH, salinity, temperature, and organic matter content or even toxic contaminants of water may affect yeast quantity and species composition. Monapathi et al. [84] found that yeasts in fresh water consist of *Candida*, *Clavispora*, *Cyberlindnera*, *Cryptococcus*, *Debaryomyces*, *Hanseniaspora*, *Kluyveromyces*, *Metschnikowia*, *Meyerozyma*, *Pichia*, *Rhodotorula*, *Saccharomyces*, *Torulaspora*, *Trichosporon*, and *Yarrowia*. Rich yeasts species reflect inputs from terrestrial sources such as soil and plant debris and anthropogenic activities. Since duckweed is an aquatic plant found on water surfaces, yeast species associated with duckweed may come from a terrestrial source to colonize duckweed. Generally, ascomycetous yeasts are most likely found in areas that tend to be rich in organic carbon, while basidiomycetous yeasts most likely use a broader range of carbon compounds at lower concentrations [85]. Therefore, the plant species may affect the occurrence of yeasts. This may be a consequence of the nutrient composition of each plant. In this case, the yeast community may be affected by the nutrient composition of duckweed.

It is notable that many duckweed associated yeasts isolated in this work have been reported as human and opportunistic pathogens, *viz*. *Candida albicans* [86], *C. metapsilosis*, *C. orthopsilosis*, *C. parapsilosis* [87],[88], *C. palmioleophila* [89], *C. tropicalis* [90], *Lodderomyces elongisporus* [91], and *Kodamaea ohmeri* [92]. These species may have come from either humans or human activities around duckweed since these samples were collected near urban areas. Glushakova et al. [93] reported the presence of pathogenic and opportunistic yeast species, *C. albicans*, *C. glabrata*, and *C. parapsilosis*, on the pollen of wind pollinated plants in the urban environment while these yeasts were not present on plant pollen in a forest. The same research group reported the presence of an opportunistic species, *C. parapsilosis*, from the internal tissue of apple and pear fruits during the entire period of fruit formation and the development is due to anthropogenic impacts in a city [45].

In the present study, the most prevalent species was the basidiomycetous yeast *Papiliotrema laurentii*, as evident by its highest relative of frequency (21.8%) and frequency of occurrence (25%). *Pa. laurentii* was reported as an endophytic yeast on corn leaf tissue [48]. This yeast was also found in plant materials such as tree bark and decaying fruits [94], decaying tree samples in India [95], decaying organic material collected from both primary and secondary peat swamp forests in Thailand [96], and soil [97]. This information implies that *Pa. laurentii* is commonly found in plant material samples such as duckweed. Moreover, *Pa. laurentii* has been reported to produce polyamines and promote root growth of the medicinal plant, *Agathosma betulina* (Berg.) Pillans [98], and to accumulate lipid for biofuel production [97],[99].

Duckweed associated yeast diversity was investigated in this study using a culture-dependent methodology, which may not have isolated all of yeast species present. However, this methodology for isolating yeasts is well adopted and reported [48],[100],[101] to assure isolation of as many yeasts as possible. The species richness estimators, Chao1, Jack1, and Bootstrap (Figure 5), showed fewer observed species than expected, indicating that some yeast species may not have been recovered. These results are in accordance with the species composition of the yeast communities associated with plants inferred by previously reported culture-dependent and culture-independent approaches [48],[73],[102],[103]. Culture-independent methods, such as using next generation sequencing (NGS) combined with a culture-dependent approach to archive complete information, may be used to study yeast diversity on duckweed. However, culture-dependent methods are beneficial in terms of yielding pure culture microorganisms that are useful bioresources for basic studies and valuable applications.

Moreover, yeasts strain obtained from natural habitats have been used to produce biochemical products. Some of the yeast species obtained from this study have been reported to have industrial biotechnological potential. For example, *Metschnikowia koreensis* was reported as carbonyl reductase producer [104]. *Cyberlindnera subsufficiens* has capabilities to produce ethanol, IAA, and extracellular enzymes [96],[105]. *M. saccharicola* was reported to produce a toxin that is lethal to yeasts that are pathogenic to crabs [62],[106]. *Cy. jadinii* was used as a source of single cell proteins [107]. *Candida ethanolica* has been reported as an antagonistic yeast against bacterial wilt disease of tomato plants [108]. Endophytic yeasts were reported as IAA producers, including *Hanseniaspora uvarum*, *Meyerozyma caribbica*, *Rhodosporidiobolus fluvialis*, and *Rhodotorula mucilaginosa*. *Candida* and *Kluyveromyces* species have been reported to produce alkaline proteases that inhibit phytopathogenic fungi [109],[110]. In the present study, IAA and siderophore production, as well as phosphate solubilization, were also observed in yeasts obtained (Table 2). This result is accordance with the report of Nutaratat et al. [25], which showed that epiphytic and endophytic yeasts isolated from rice and sugar cane leaves in Thailand produced plant growth promoting factors such as IAA and siderophores, as well as phosphate and zinc solubilization, 1-aminocyclopropane-1-carboxylate (ACC) deaminase activity, and antagonistic activity against fungal rice pathogens. Furthermore, many plants associated with yeasts were reported to possess plant growth promoting capabilities. For example, endophytic yeasts from agriculturally grown fruits produced IAA [100], while endophytic yeasts from strawberry (*Fragaria* × *ananassa*) leaves and wheat (*Triticum aestivum*) seeds were positively identified for phosphate solubilization, siderophore production, proteolytic activity, and ammonia production [111]. The diversity of ascomycetous and basidiomycetous yeast species from duckweed may lead to potential and valuable applications.

*Pichia* has been reported as a plant growth promoting yeast [112]. The study of phosphate solubilizing fungi isolated and characterized from Teff (*Eragrostis teff*) rhizosphere soil, collected from the North Shewa and Gojam, Ethiopia, reported that *P. norvegensis* had phosphate solubilizing capability [113]. *Pichia* sp. CC1 also showed an ability to promote lettuce growth by increasing the availability of phosphorus in the soil [114]. However, there has been no report of *P. kluyveri* with phosphate solubilization capability.

## 5. Conclusions

A culture-dependent method was used to study the diversity of yeasts on duckweed (*Lemnaceae*). Yeast identification was based on the D1/D2 region of the large subunit (LSU) rRNA gene sequence analysis. The results revealed that of the yeasts associated with duckweed, 55.2% were ascomycetous and 44.8% were basidiomycetous. The basidiomycetous yeast *Papiliotrema laurentii* was identified as the most prevalent species, with a relative of frequency and frequency of occurrence of 21.8% and 25%, respectively. High values of diversity indices were shown in this study, as indicated by the Shannon-Wiener index (*H′*), Shannon equitability index (*E_H_*), and Simpson diversity index (*1-D*) values of 3.48, 0.86, and 0.96, respectively. The present study revealed that the yeast community on duckweed shows high diversity and evenness of species. The study of plant growth promoting traits of yeasts from duckweed revealed that 178 yeast strains produced IAA in the range of 0.08–688.93 mg/L. One hundred and six strains showed phosphate solubilization activity in range of 0.32–2.13 solubilization efficiency (SE) units and 173 yeast strains produced siderophores in range of 0.94–2.55 siderophore activity units (AU). This work indicates that duckweed is a potential resource to obtain plant growth promoting yeasts.
