# Supplementary material for: Diversity of duckweed (Lemnaceae) associated yeasts and their plant growth promoting characteristics
Source: AIMS Microbiol. 2023 May 16;9(3):486–517. doi: 10.3934/microbiol.2023026 (PMC10462456; doi:10.3934/microbiol.2023026)
Supplement: Supplementary file 1 [file microbiol-09-03-026-s001.pdf]

AIMS Microbiology

AIMS Microbiology, 9(3): 486–517.
DOI: 10.3934/microbiol.2023026
Received: 23 March 2023
Revised: 02 May 2023
Accepted: 09 May 2023
Published:16 May 2023

http://www.aimspress.com/journal/microbiology

Research article

Diversity of duckweed (*Lemnaceae*) associated yeasts and their plant growth promoting characteristics

Napapohn Kajadpai[1], Jirameth Angchuan[1], Pannida Khunnamwong[1,2] and Nantana Srisuk[1, 2,*]

[1] Department of Microbiology, Faculty of Science, Kasetsart University, Bangkok, 10900, Thailand
[2] Biodiversity Center Kasetsart University (BDCKU), Bangkok 10900, Thailand

* **Correspondence:** Email: fscints@ku.ac.th; Tel: +6625625444; Ext: 6466114; Fax: +6625792081.

Supplementary

Table S1. Duckweed samples and number of isolated yeast strains in this study.

Date of sampling	Sample code	Province	GPS coordinates	Genus of duckweed	Number of isolated yeasts	Yeast strain code
6/2/2021	DW1	Bangkok	13°50'39.9"N/100°34'15.6"E	*Lemna*	3	DW1-1,2,3
15/2/2021	DW2	Bangkok	-	*Spirodela*	1	DW2-1
15/2/2021	DWW1	Bangkok	-	*Lemna*	2	DWW1-8,9
15/2/2021	DWW2	Bangkok	-	*Wolffia*	4	DWW2-1,2,3,4
20/2/2021	DW3	Samutsongkhram	13°28'04.6"N/99°53'38.4"E	*Lemna*	8	DW3-1,2,3,4,5,7,8,9
4/3/2021	DW4	Bangkok	13°51’31.1”N/100°40’31.4”E	*Lemna*	2	DW4-1,2
11/3/2021	DW5	Bangkok	13°50'58.0"N/100°34'07.7"E	*Lemna*	0	-
14/3/2021	DW6	Suphanburi	14°25'43.0"N/100°00'28.8"E	*Spirodela*	0	-
14/3/2021	DW7	Suphanburi	14°25'39.0"N/100°00'50.4"E	*Lemna*	1	DW7-1
14/3/2021	DW8	Suphanburi	14°25'35.7"N/100°00'53.5"E	*Lemna*	3	DW8-2,3,4
26/3/2021	DW9	Bangkok	13°52'36.8"N/100°40'55.3"E	*Spirodela*	0	-
26/3/2021	DW11	Pathumthani	13°55'52.2"N/100°44'49.5"E	*Spirodela*	0	-

Continued on next page

Date of sampling	Sample code	Province	GPS coordinates	Genus of duckweed	Number of isolated yeasts	Yeast strain code
26/3/2021	DW12	Pathumthani	13°55'52.2"N/100°44'49.5"E	*Spirodela*	0	-
26/3/2021	DW13	Pathumthani	13°55'52.2"N/100°44'49.5"E	*Wolffia*	0	-
26/3/2021	DW14	Pathumthani	13°55'51.1"N/100°44'45.4"E	*Lemna*	0	-
26/3/2021	DW15	Pathumthani	13°55'46.6"N/100°43'58.3"E	*Lemna*	2	DW15-1,2
26/3/2021	DW16	Bangkok	13°54'01.5"N/100°41'33.9"E	*Lemna*	0	-
2/4/2021	DWW14 S	-	-	*Spirodela*	8	DWW14 S-1,2,3,4,5,6,7,8
5/4/2021	DWW14 W	-	-	*Wolffia*	4	DWW14 W-1,2,3,4
25/8/2021	DW18	Bangkok	13°51'03.3"N/100°34'38.1"E	*Spirodela*	2	DW18-1,2
3/9/2021	DWW3	Chumphon	10°32'37.3"N/99°11'53.1"E	*Spirodela*	8	DWW3-1,2,3,4,5,6,7,8
3/9/2021	DWW4	Chumphon	10°32'37.3"N/99°11'53.1"E	*Lemna*	8	DWW4-1,2,3,4,6,7,8,9
3/9/2021	DW20	Bangkok	13°50'53.2"N/100°33'59.8"E	*Spirodela*	0	-
19/9/2021	DWEN21	Bangkok	13°77'53.8"N/100°75'88.6"E	*Lemna*	2	DWEN21-1,2
19/9/2021	DWEN22	Bangkok	13°77'54.3"N/100°75'95.6"E	*Lemna*	8	DWEN22-1,2,3,4,5,6,7,8
19/9/2021	DWEN23	Bangkok	13°78'02.6"N/100°76'11.8"E	*Lemna*	9	DWEN23-1,2,3,4,5,6,7,8,9
19/9/2021	DWEN24	Bangkok	13°78'03.6"N/100°76'11.4"E	*Lemna*	0	-
4/10/2021	DWW5	Prachuap Khiri Khan	12°07'31.3"N/99°54'20.4"E	*Lemna*	11	DWW5-1,2,3,4,5,6,7,8,9,11,12
4/10/2021	DWW6	Prachuap Khiri Khan	12°07'31.3"N/99°54'20.4"E	*Spirodela*	1	DWW6-1
4/10/2021	DWW11	Prachuap Khiri Khan	12°07'31.3"N/99°54'20.4"E	*Wolffia*	2	DWW11-1,2
18/10/2021	DWEN25	Bangkok	13°46'33.5"N/100°46'06.6"E	*Lemna*	0	-
18/10/2021	DWEN26	Bangkok	13°46'33.0"N/100°46'07.8"E	*Lemna*	7	DWEN26-3,4,5,6,7,9,10
18/10/2021	DWEN27	Bangkok	13°46'43.9"N/100°46'41.1"E	*Lemna*	2	DWEN27-1,2
3/11/2021	DWW7	Prachuap Khiri Khan	12°14'00.9"N/99°55'40.2"E	*Landotia*	1	DWW7-1
4/11/2021	DWW8	Prachuap Khiri Khan	12°14'37.1"N/99°55'57.0"E	*Lemna*	1	DWW8-1
5/11/2021	DWW9	Prachuap Khiri Khan	12°23'03.0"N/99°59'01.9"E	*Lemna*	1	DWW9-1

Continued on next page

Date of sampling	Sample code	Province	GPS coordinates	Genus of duckweed	Number of isolated yeasts	Yeast strain code
6/11/2021	DWW10	Prachuap Khiri Khan	12°56'52.7"N/99°55'55.3"E	*Lemna*	3	DWW10-2,3,4
12/11/2021	DWEN28	Bangkok	13°51'13.0"N/100°40'26.9"E	*Lemna*	2	DWEN28-1,2
12/11/2021	DWEN29	Bangkok	13°52'07.9"N/100°41'28.2"E	*Lemna*	12	DWEN29-1,2,3,4,5,6,7,8,9,10,11,12
12/11/2021	DWEN30	Bangkok	13°51'36.1"N/100°40'32.6"E	*Lemna*	16	DWEN30-1,2,3,4,5,6,7,8,9,10,11,12,13,14,15,16
15/11/2021	DWEN31	Pathumthani	13°57'03.1"N/100°49'07.0"E	*Lemna*	3	DWEN31-1,2,3
15/11/2021	DWEN32	Pathumthani	13°56'59.2"N/100°48'48.6"E	*Spirodela*	3	DWEN32-1,2,3
15/11/2021	DWEN33	Nakornnayok	14°06'53.6"N/100°59'09.3"E	*Lemna*	6	DWEN33-1,2,3,5,6,8
15/11/2021	DWEN34	Prajinburi	13°58'29.4"N/101°08'56.9"E	*Lemna*	6	DWEN34-2,3,4,5,6,7
15/11/2021	DWEN35	Sa Kaew	13°26'25.8"N/102°08'00.6"E	*Spirodela*	4	DWEN35-1,2,3,4
16/11/2021	DWEN36	Trad	12°11'10.3"N/102°29'10.2"E	*Lemna*	8	DWEN36-1,2,3,4,5,6,7,8
16/11/2021	DWEN37	Rayong	12°47'54.8"N/101°39'52.8"E	*Lemna*	10	DWEN37-1,2,4,5,6,7,8,9,10,11
17/11/2021	DWEN38	Chonburi	13°13'22.4"N/100°58'02.7"E	*Landotia*	8	DWEN38-1,2,4,5,6,7,8,9
17/11/2021	DWEN39	Chachoengsao	13°33'49.2"N/100°59'49.6"E	*Lemna*	6	DWEN39-1,2,3,4,5,6
17/11/2021	DWEN40	Chachoengsao	13°35'12.5"N/101°04'04.1"E	*Lemna*	0	-
9/4/2022	DWEN41	Bangkok	13°51'51.8"N/100°45'32.6"E	*Landotia*	4	DWEN41-1,2,3,4
15/4/2022	DWEN42	Bangkok	13°47'19.8"N/100°49'36.2"E	*Landotia*	1	DWEN42-1
15/4/2022	DWEN43	Bangkok	13°46'57.5"N/100°49'46.9"E	*Landotia*	2	DWEN43-1,2
15/4/2022	DWEN44	Bangkok	13°46'57.2"N/100°49'47.0"E	*Landotia*	0	-
15/4/2022	DWEN45	Bangkok	13°45'33.7"N/100°50'19.5"E	*Landotia*	1	DWEN45-1
15/4/2022	DWEN46	Bangkok	13°45'27.1"N/100°50'19.9"E	*Spirodela*	0	-
15/4/2022	DWEN47	Bangkok	13°45'30.0"N/100°50'19.8"E	*Landotia*	1	DWEN47-1
15/4/2022	DWEN48	Bangkok	13°45'15.6"N/100°50'21.6"E	*Lemna*	0	-
15/4/2022	DWEN49	Bangkok	13°45'25.6"N/100°50'19.9"E	*Lemna*	2	DWEN49-1,2
24/4/2022	DWEN50	Kanchanaburi	14°03'45.5"N99°52'77.6"E	*Lemna*	1	DWEN50-1
24/4/2022	DWEN51	Kanchanaburi	14°03'44.9"N/99°52'78.1"E	*Lemna*	0	-

Continued on next page

Date of sampling	Sample code	Province	GPS coordinates	Genus of duckweed	Number of isolated yeasts	Yeast strain code
24/4/2022	DWEN52	Kanchanaburi	14°03'40.4"N/99°52'81.4"E	*Lemna*	0	-
3/5/2022	DWEN53	Nonthaburi	13°99'59.6"N/100°30'11.2"E	*Landotia*	5	DWEN53-1,2,3,4,5
3/5/2022	DWEN54	Nonthaburi	14°00'00.3"N/100°27'43.1"E	*Lemna*	3	DWEN54-2,3,6
3/5/2022	DWEN55	Nakhon Pathom	14°01'08.5"N/100°25'98.3"E	*Lemna*	5	DWEN55-1,2,3,4,6
3/5/2022	DWEN56	Nakhon Pathom	14°00'78.9"N/100°24'32.6"E	*Lemna*	9	DWEN56-1,2,3,5,6,7,9,10,11
3/5/2022	DWEN57	Nakhon Pathom	14°01'53.9"N/100°19'28.9"E	*Landotia*	0	-
3/5/2022	DWEN58	Nakhon Pathom	14°01'36.0"N/100°15'75.2"E	*Lemna*	0	-
3/5/2022	DWEN59	Nakhon Pathom	14°01'35.2"N/100°15'75.7"E	*Lemna*	8	DWEN59-1,2,3,4,5,6,7,8
3/5/2022	DWEN60	Nakhon Pathom	14°01'33.8"N/100°15'69.4"E	*Spirodela*	5	DWEN60-1,2,3,4,5
3/5/2022	DWEN61	Nakhon Pathom	14°01'25.2"N/100°15'73.1"E	*Spirodela*	4	DWEN61-1,2,3,5
3/5/2022	DWEN62	Nakhon Pathom	14°00'91.6"N/100°23'68.9"E	*Lemna*	13	DWEN62-1,2,3,4,5,6,7,8,9,10,11,12,13

-; Data not available

Table S2. Number of strains, relative frequency and frequency of occurrence of yeasts isolated from duckweed samples.

Yeast taxa	No. of yeast strains from duckweed genera					RF (%)	FO (%)
	Landotia	*Lemna*	*Spirodella*	*Wolffia*			
Phylum Ascomycota					139		
Subphylum Saccharomycotina							
Debaryomycetaceae							
Candida albicans (*Candida/Lodderomyces* clade)	nd	1	nd	nd	1	0.4	1.4
Candida jaroonii (*Yamadazyma* clade)	nd	1	nd	nd	1	0.4	1.4
Candida metapsilosis (*Candida/Lodderomyces* clade)	nd	1	nd	nd	1	0.4	1.4
Candida orthopsilosis (*Candida/Lodderomyces* clade)	nd	nd	nd	4	4	1.6	1.4
Candida palmioleophila (*Candida glaebosa* clade)	nd	5	nd	nd	5	2	2.8
Candida parapsilosis (*Candida/Lodderomyces* clade)	nd	9	nd	nd	9	3.6	5.6
Candida tropicalis (*Candida/Lodderomyces* clade)	1	16	1	nd	19	7.5	15.3
Candida sp. group 1	nd	1	nd	nd	1	0.4	1.4
(closely related to *C. tropicalis* in *Candida/Lodderomyces* clade)							
Debaryomyces singareniensis	1	nd	1	nd	1	0.4	1.4
Lodderomyces elongisporus	nd	1	nd	nd	1	0.4	1.4
Meyerozyma caribbica	2	7	2	nd	11	4.4	9.7
Meyerozyma carpophila	nd	4	1	nd	5	2	5.6
**Metschnikowiaceae**							
Candida pseudointermedia (*Candida*/*Metschnikowiaceae* clade)	nd	3	nd	nd	3	1.2	4.2
Candida sp. group 2	nd	2	nd	nd	2	0.8	1.4
(closely related to *C. suratensis*							
in *Candida/Metschnikowiaceae* clade)							
Kodamaea ohmeri	2	3	2	nd	5	2	4.2
Metschnikowia koreensis	nd	1	nd	nd	1	0.4	1.4
Metschnikowia saccharicola	nd	nd	2	nd	2	0.8	1.4

Yeast taxa	No. of yeast strains from duckweed genera					RF (%)	FO (%)
	Landotia	*Lemna*	*Spirodella*	*Wolffia*			
**Phaffomycetaceae**							
Candida sp. group3 (closely related to *Candida yuanshanica* in *Candida/Wickerhamomyces* clade)	nd	nd	1	nd	1	0.4	1.4
Cyberlindnera fabianii	nd	2	nd	nd	2	0.8	1.4
Cyberlindnera jadinii	nd	nd	1	nd	1	0.4	1.4
Cyberlindnera subsufficiens	nd	nd	2	nd	2	0.8	1.4
Starmera stellimalicola	nd	1	nd	nd	1	0.4	1.4
**Pichiaceae**							
Candida ethanolica (Candida/Pichia clade*)*	nd	3	nd	nd	3	1.2	1.4
Candida pseudolambica (Candida/Pichia clade*)*	nd	5	2	nd	7	2.8	8.3
Candida sp. group 4 (closely related to *Candida pseudolambica in Candida/Pichia* clade)	nd	1	nd	nd	1	0.4	1.4
Ogataea thermomethanolica	nd	1	nd	nd	1	0.4	1.4
Pichia kluyveri	nd	nd	1	nd	1	0.4	1.4
Pichia kudriavzevii	nd	12	2	1	15	6	12.5
Pichia manshurica	nd	nd	1	nd	1	0.4	1.4
Pichia occidentalis	nd	nd	1	nd	1	0.4	1.4
**Saccharomycetaceae**							
Kluyveromyces marxianus	nd	2	nd	nd	2	0.8	1.4
Kluyveromyces starmeri	nd	2	1	nd	3	1.2	2.8
**Saccharomycetales** *incertae sedis*							
Candida nonsorbophila (*Candida*/*Saccharomycetales* clade)	nd	1	nd	nd	1	0.4	1.4
Crinitomyces flavificans	1	8	2	1	12	4.8	16.7
Diutina rugosa	1	1	nd	nd	2	0.8	2.8

Yeast taxa	No. of yeast strains from duckweed genera					RF (%)	FO (%)
	Landotia	*Lemna*	*Spirodella*	*Wolffia*			
Sporopachydermia lactativora	nd	1	nd	nd	1	0.4	1.4
Starmerella sp. (closely related to *Starmerella caucasica*)	nd	1	2	nd	3	1.2	2.8
**Saccharomycodaceae**							
Hanseniaspora opuntiae	nd	1	nd	nd	1	0.4	1.4
**Trichomonascaceae**							
Wickerhamiella infanticola	nd	3	nd	nd	3	1.2	4.2
Wickerhamiella martinezcruziae	nd	1	nd	nd	1	0.4	1.4
Zygoascus sp. (closely related to *Zygoascus polysorbophila*)	nd	1	nd	nd	1	0.4	1.4
**Phylum Basidiomycota**					**113**		
**Subphylum Agaricomycotina**							
**Filobasidiaceae**							
Naganishia liquefaciens	1	nd	nd	nd	1	0.4	1.4
**Cryptococcaceae**							
Kwoniella heveanensis	nd	1	nd	nd	1	0.4	1.4
**Rhynchogastremaceae**							
Papiliotrema aspenensis	nd	1	nd	nd	1	0.4	1.4
Papiliotrema laurentii	5	41	7	2	55	21.8	25
Papiliotrema rajasthanensis	nd	1	nd	nd	1	0.4	1.4
Papiliotrema ruineniae	1	nd	nd	nd	1	0.4	1.4
Papiliotrema sp. (closely related to *Papiliotrema laurentii*)	nd	2	3	nd	5	2	6.9
**Trichosporonaceae**							
Apiotrichum loubieri	nd	1	nd	nd	1	0.4	1.4
**Subphylum Pucciniomycotina**							
**Sporidiobolaceae**							
Rhodosporidiobolus fluvialis	nd	nd	2	2	4	1.6	2.8
Rhodosporidiobolus ruineniae	nd	6	nd	nd	6	2.4	1.4

Yeast taxa	No. of yeast strains from duckweed genera					RF (%)	FO (%)
	Landotia	*Lemna*	*Spirodella*	*Wolffia*			
Rhodotorula mucilaginosa	4	12	nd	nd	16	6.3	13.9
Rhodotorula paludigena	nd	1	nd	nd	1	0.4	1.4
Rhodotorula taiwanensis	1	9	1	nd	11	4.4	8.3
Rhodotorula diobovata	nd	2	nd	nd	2	0.8	1.4
Rhodotorula sp. (closely related to *Rhodotorula toruloides*)	nd	1	nd	nd	1	0.4	1.4
**Subphylum Ustilaginomycotina**							
**Ustilaginaceae**							
Moesziomyces antarcticus	nd	1	3	nd	4	1.6	4.2
Pseudozyma churashimaensis	nd	2	nd	nd	2	0.8	1.4

RF; Relative frequency (%) was calculated as the number of isolates of a particular species as a proportion of the total number of strains.

FO; Frequency of occurrence (%) was calculated as number of samples, where a particular species was observed as a proportion of the total number of samples.

nd; not detected.

Table S3. Identification of endophytic yeasts from duckweed *(Lemnaceae)* based on analysis of the sequences of the D1/D2 region

Taxa	No. of strain	Strain DMKU-	D1/D2 GenBank accession no. of closest species	Similarity to closest species (%)	**No. of sample**
**Phylum Ascomycota**					
**Subphylum Saccharomycotina**					
**Debaryomycetaceae**					
Candida albicans (*Candida/Lodderomyces* clade)	1	DWEN59-3	NG054826	100	1
Candida jaroonii (*Yamadazyma* clade)	1	DWW4-4	AB292057	100	1
Candida metapsilosis (*Candida/Lodderomyces* clade)	1	DWEN56-2	NG054815	99.82	1
Candida orthopsilosis (*Candida/Lodderomyces* clade)	4	DWW2-1, DWW2-2, DWW2-3, DWW2-4	NG054816	99.82-100	1
Candida palmioleophila (*Candida glaebosa* clade)	5	DWW30-4, DWW30-5, DWW30-6, DWW30-13, DWEN56-10	KY106645	100	2
Candida parapsilosis (*Candida/Lodderomyces* clade)	9	DW8-4, DWEN30-3, DWEN30-7, DWEN30-9, DWEN30-10, DWEN30-15, DWEN39-1, DWEN39-2, DWEN62-9	NG054833	100	4
Candida tropicalis (*Candida/Lodderomyces* clade)	19	DWW4-2, DW18-1, DW18-2, DWEN23-4, DWEN23-6, DWEN23-7, DWEN23-8, DWEN29-1, DWEN30-2, DWEN30-8, DWEN30-14, DWEN30-16, DWEN33-3, DWEN38-1, DWEN49-1 DWEN54-2, DWEN56-1, DWEN62-1, DWEN62-6	NG054834	99.82-100	11
Candida sp. group 1 (closely related to *C. tropicalis* in *Candida/Lodderomyces* clade)	1	DWEN39-5	NG054834	99.11	1
Debaryomyces singareniensis	1	DWEN45-1	KY107585	100	1

Continued on next page

Taxa	No. of strain	Strain DMKU-	D1/D2 GenBank accession no. of closest species	Similarity to closest species (%)	**No. of sample**
Lodderomyces elongisporus	1	DWW1-9	KY108330	100	1
Meyerozyma caribbica	11	DWEN23-1, DWEN23-9, DWEN34-2, DWEN34-4, DWEN34-5, DWEN34-6, DWEN43-1, DWEN53-2, DWEN60-3, DWEN61-2, DWEN62-11	NG054806	100	7
Meyerozyma carpophila	5	DWW3-4, DWW4-1, DWW4-9, DW3-1 DWEN39-4	NG069408	99.64-100	4
**Metschnikowiaceae**					
Candida pseudointermedia (*Candida*/*Metschnikowiaceae* clade)	3	DWEN30-1, DWEN34-7, DWEN62-8	NG055407	99.8-100	3
Candida sp. group 2 (closely related to *Candida suratensis* in *Candida/Metschnikowiaceae* clade)	2	DW3-4, DW3-8	AB500863	98.80-99.20	1
Kodamaea ohmeri	5	DWEN23-3, DWEN23-5, DWEN38-6, DWEN38-9, DWEN50-1	MK394144	99.79-100	3
Metschnikowia koreensis	1	DWEN23-3	NG058340	99.6	1
Metschnikowia saccharicola	2	DWEN35-1, DWEN35-3	AB697755	99.57	1
**Phaffomycetaceae**					
Candida sp. group 3 (closely related to *Candida yuanshanica* in *Candida/Wickerhamomyces* clade)	1	DWEN35-4	NG057184	98.67	1
Cyberlindnera fabianii	2	DWEN28-1, DWEN28-2	NG055731	100	1
Cyberlindnera jadinii	1	DWW14 S-2	NG056278	99.41	1
Cyberlindnera subsufficiens	2	DWW3-2, DWW3-8	NG059002	99.76-99.82	1

Continued on next page

Taxa	No. of strain	Strain DMKU-	D1/D2 GenBank accession no. of closest species	Similarity to closest species (%)	**No. of sample**
Starmera stellimalicola	1	DWEN23-2	NG060829	100	1
**Pichiaceae**					
Candida ethanolica (Candida/Pichia clade)	3	DWEN62-10, DWEN62-12, DWEN62-13	NG055105	100	1
Candida pseudolambica (Candida/Pichia clade)	7	DWW3-1, DWW10-2, DWW10-4, DWEN26-3, DWEN35-2, DWEN39-3, DWEN49-2	NG060822	99.45-99.81	6
Candida sp. group 4 (closely related to *Candida pseudolambica* in *Candida/Pichia* clade)	1	DWEN34-3	NG060822	99.06	1
Ogataea thermomethanolica	1	DWW4-8	AB200285	99.42	1
Pichia kluyveri	1	DWEN38-8	NG055122	99.82	1
Pichia kudriavzevii	15	DWW10-3, DWW11-1, DWEN26-7, DWEN54-3, DWEN56-7, DWEN56-11, DWEN59-4, DWEN59-5, DWEN59-6, DWEN59-8, DWEN60-1, DWEN61-1, DWEN62-2, DWEN62-5, DWEN62-7	NG055104	100	9
Pichia manshurica	1	DWEN59-7	KY108860	99.82	1
Pichia occidentalis	1	DW1-1	NG055110	100	1
**Saccharomycetaceae**					
Kluyveromyces marxianus	2	DWW5-1, DWW5-11	JQ689023	100	1
Kluyveromyces starmeri	3	DW2-1, DWEN26-4, DWEN26-5	KT853036	100	2
**Saccharomycetales** *incertae sedis*					
Candida nonsorbophila (*Candida/Saccharomycetales* clade)	1	DWEN26-9	NG055173	100	1

Continued on next page

Taxa	No. of strain	Strain DMKU-	D1/D2 GenBank accession no. of closest species	Similarity to closest species (%)	**No. of sample**
Crinitomyces flavificans	12	DWW3-3, DWW3-6, DWW4-7, DWW5-3, DWW5-6, DWW5-8, DWW5-9, DWW5-12, DWW7-1, DWW8-1, DWW11-2, DW1-3	MH873011	99.80-100	7
Diutina rugosa	2	DWEN26-6, DWEN53-3	GU246244	99.77-88.78	2
Sporopachydermia lactativora	1	DWW5-7	NG042647	100	1
Starmerella sp. (closely related to *Starmerella caucasica*)	3	DWEN31-1, DWEN32-2, DWEN32-3	NG058407	97.26-97.42	2
**Saccharomycodaceae**					
Hanseniaspora opuntiae	1	DWEN27-1	NG055312	100	1
**Trichomonascaceae**					
Wickerhamiella infanticola	3	DW1-2, DW3-3, DWEN30-12	NG058278	99.53-100	3
Wickerhamiella martinezcruziae	1	DWEN62-4	KM246828	99.45	1
Zygoascus sp. (closely related to *Zygoascus polysorbophila*)	1	DW7-1	NG064312	99.03	1
**Phylum Basidiomycota**					
**Subphylum Agaricomycotina**					
**Filobasidiaceae**					
Naganishia liquefaciens	1	DWEN47-1	NG057655	100	1
**Cryptococcaceae**					
Kwoniella heveanensis	1	DWEN33-2	NG057655	100	1
**Rhynchogastremaceae**					
Papiliotrema aspenensis	1	DWEN36-2	NG060109	100	1

Continued on next page

Taxa	No. of strain	Strain DMKU-	D1/D2 GenBank accession no. of closest species	Similarity to closest species (%)	**No. of sample**
Papiliotrema laurentii	55	DWW1-8,DWW9-1, DWW14 S-1, DWW14 S-2, DWW14 S-3, DWW14 S-8, DWW14 W-2, DWW14 W-3, DW8-3, DW15-1, DW15-2, DWEN21-1, DWEN21-2, DWEN21-3, DWEN21-4, DWEN22-5, DWEN22-6, DWEN22-7, DWEN22-8, DWEN29-2, DWEN29-4, DWEN29-5, DWEN29-6, DWEN29-7, DWEN29-8, DWEN29-9, DWEN29-10, DWEN29-11, DWEN29-12, DWEN31-1, DWEN31-2, DWEN31-3, DWEN36-1, DWEN36-3, DWEN36-4, DWEN36-5, DWEN36-6, DWEN36-7, DWEN36-8, DWEN37-1, DWEN37-4, DWEN37-7, DWEN37-8, DWEN37-9, DWEN37-10, DWEN38-4, DWEN38-5, DWEN38-7, DWEN41-1, DWEN41-2, DWEN41-4, DWEN54-6, DWEN55-1, DWEN60-2, DWEN60-4, DWEN61-3	NG056281	99.60-99.83	18
Papiliotrema rajasthanensis	1	DWEN30-11	NG058366	100	1
Papiliotrema ruineniae	1	DWEN53-4	LK023764	100	1
Papiliotrema sp. (closely related to *Papiliotrema laurentii*)	5	DWW14 S-6, DWEN29-3, DWEN55-2, DWEN60-5, DWEN61-5	NG056281	99.46-99.49	5
**Trichosporonaceae**					
Apiotrichum loubieri	1	DWEN27-2	KY106133	100	1
**Subphylum Pucciniomycotina**					

Continued on next page

Taxa	No. of strain	Strain DMKU-	D1/D2 GenBank accession no. of closest species	Similarity to closest species (%)	**No. of sample**
**Sporidiobolaceae**					
Rhodosporidiobolus fluvialis	4	DWW14 S-4, DWW14 S-7, DWW14 W-1, DWW14 W-4	NG042341	99.62	2
Rhodosporidiobolus ruineniae	6	DW8-2, DWEN26-10, DWEN33-1, DWEN33-5, DWEN33-6, DWEN33-8	KY108979	99.81-100	3
Rhodotorula mucilaginosa	16	DW3-5, DW3-7, DW4-1, DW4-2, DWEN22-1, DWEN22-2, DWEN39-6, DWEN42-1, DWEN43-2, DWEN53-1, DWEN53-5, DWEN56-3, DWEN56-6, DWEN56-9, DWEN59-1, DWEN62-3	KY109056	99.82-100	10
Rhodotorula paludigena	1	DWW4-6	NG042383	99.8	1
Rhodotorula taiwanensis	11	DWW5-5, DWW14 S-5, DWEN37-2, DWEN37-5, DWEN37-6, DWEN37-11, DWEN41-3, DWEN55-3, DWEN55-4, DWEN55-6, DWEN56-5	KY109163	99.79-100	6
Rhodotorula diobovata	2	DW3-1, DW3-9	NG042340	99.81	1
Rhodotorula sp. (closely related to *Rhodotorula toruloides*)	1	DWEN59-2	KY109167	99.43	1
**Subphylum Ustilaginomycotina**					
**Ustilaginaceae**					
Moesziomyces antarcticus	4	DWW3-5, DWW3-7, DWW4-3, DWW6-1	KY108571	100	3
Pseudozyma churashimaensis	2	DWW5-2, DWW5-4	AB548955	100	1

AIMS Press

© 2023 the Author(s), licensee AIMS Press. This is an open access article distributed under the terms of the Creative Commons Attribution License (http://creativecommons.org/licenses/by/4.0)
